# Zearalenone contamination in maize, its associated producing fungi, control strategies, and legislation in Sub‐Saharan Africa

**DOI:** 10.1002/fsn3.4125

**Published:** 2024-04-17

**Authors:** Abdul Rashid Hudu, Francis Addy, Gustav Komla Mahunu, Abdul‐Halim Abubakari, Nelson Opoku

**Affiliations:** ^1^ Department of Agricultural Biotechnology, Faculty of Agriculture, Food and Consumer Sciences University for Development Studies Nyankpala Ghana; ^2^ Department of Biotechnology and Molecular Biology, Faculty of Biosciences University for Development Studies Nyankpala Ghana; ^3^ Department of Food Science and Technology, Faculty of Agriculture, Food, and Consumer Sciences University for Development Studies Nyankpala Ghana; ^4^ Department of Horticulture, Faculty of Agriculture, Food, and Consumer Sciences University for Development Studies Nyankpala Ghana

**Keywords:** Africa, gene expression, nonthermal processing, PCR‐ELISA, zearalenone

## Abstract

The fungal genus *Fusarium* contains many important plant pathogens as well as endophytes of wild and crop plants. Globally, *Fusarium* toxins in food crops are considered one of the greatest food safety concerns. Their occurrence has become more pronounced in Africa in recent times. Among the major *Fusarium* mycotoxins with food and feed safety concerns, zearalenone is frequently detected in finished feeds and cereals in Africa. However, the impact of indigenous agricultural practices (pre‐ and postharvest factors) and food processing techniques on the prevalence rate of *Fusarium* species and zearalenone occurrence in food and feed have not been collated and documented systematically. This review studies and analyzes recent reports on zearalenone contamination in maize and other cereal products from Africa, including its fungi producers, agronomic and climate variables impacting their occurrences, preventive measures, removal/decontamination methods, and legislations regulating their limits. Reports from relevant studies demonstrated a high prevalence of *F*. *verticillioides* and *F*. *graminearum* as Africa's main producers of zearalenone. Elevated CO_2_ concentration and high precipitation may carry along an increased risk of zearalenone contamination in maize. African indigenous processing methods may contribute to reduced ZEA levels in agricultural products and foods. Most African countries do not know their zearalenone status in the food supply chain and they have limited regulations that control its occurrence.

## Graphical abstract

Although maize has a significant contribution to human nutrition, the grain is susceptible to *Fusarium* infection and ZEA contamination due to poor pre‐ and post‐harvest factors. However, there are noticeable postharvest measures and processing factors that can reduce ZEA concentration in processed foods. Evidence of high dietary exposure in Africa is limited for use in the establishment of maximum permitted levels in food and feed raw material.

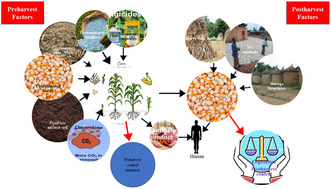

## 1 INTRODUCTION

*Fusarium* species are widely distributed and are well known as one of the world's most damaging fungi pathogens. The most harmful groups, zearalenone, trichothecenes, and fumonisin producers, continue to threaten sustainable agriculture and food safety in many regions. Recent reports have shown their common occurrence in African food and feed samples (Biomin, 2018, 2019; Gruber‐Dorninger et al., 2019). Their postharvest occurrence, coupled with pre‐ and postharvest factors such as water activity, temperature, or repeated misuse of mineral fertilizer are causing significant crop yield losses, and accumulation of mycotoxins (Mielniczuk & Skwaryło‐Bednarz, 2020). For example, excess nitrogen favored increased infestation of *Fusarium* fungi and subsequent production of zearalenone (ZEA) (Blandino et al., 2008; Podolska et al., 2017; Yi et al., 2001). Rapid infestation of grains by *Fusarium* species may cause a yield reduction of up to 19% (Lobulu et al., 2021), and severe water stress could cause the production of mycotoxins in kernels (Daou et al., 2021). As a result, foods and animal feeds are exposed to multiple mycotoxins simultaneously (Rodrigues et al., 2011). Mycotoxins are toxic metabolites of fungi that have adverse health effects on plants, animals, and humans. Among the identified *Fusarium* mycotoxins with food and feed safety concerns, ZEA is frequently detected in feeds and cereals in Africa (Gruber‐Dorninger et al., 2018). ZEA and its metabolites are the most studied mycotoxin with endocrine‐disrupting activity (Eze et al., 2018). In Africa, especially South Africa, ZEA has been associated with the clinical diagnosis of gynecomastia with testicular atrophy in males (Shephard, 2008). Its prevalence rate in agri‐food is becoming a prioritized area of research, particularly in Africa.

Limited studies on the natural occurrence of *Fusarium* species and ZEA in food and feed in Africa are available in literature (Tables 2 and 3). The effect of ZEA on human and animal health has prompted some countries to establish appropriate, permissible levels in foodstuff intended for human and animal use. In the European Union, the maximum level for ZEA ranges between 0.5 and 400 μg/kg in various products intended for human and animal consumption (European Commission, 2006a, 2006b). For example, the maximum acceptable level for ZEA in China is 60 μg/kg (Ward, 2018). Currently, few legislations (South Africa and Morocco) are available for regulating ZEA in food and feed in Africa (Ankwasa et al., 2021; Lahouar et al., 2018).

There are few comprehensive databases on the prevalence rate of *Fusarium* species and ZEA occurrence in Africa. However, these data are limited to natural with no consolidated data on the impact of traditional agronomic practices and climate change on *Fusarium* and ZEA concentrations. To facilitate data consolidation on the prevalence of *Fusarium* species and ZEA occurrence in Africa, this article highlights detailed information on ZEA and its associated producing fungi, agronomic practices, and climate change impacting their occurrence, current exposure levels, and regulatory regime in Africa. Furthermore, it summarizes stress levels leading to the production of ZEA in food and feed in Africa.

## 2 TRENDS IN MAIZE CONSUMPTION AND NUTRIENT SUPPLY IN AFRICA

Although it is clear that maize is a multipurpose crop, maize use as direct human food is repeatedly high in Africa (54%) compared to its global 56% use in feed production and 13% use as a direct human (Erenstein et al., 2022). Maize grain is the first most consumed cereal in Africa with an intake ranging from 50 to >330 g/person/day (Palacios‐Rojas et al., 2020; Ranum & Pe, 2014). The crop and its derived products including infant formulas are widely consumed in Eastern and Southern Africa, contributing to more than 30% of total calorie intake (Nuss & Tanumihardjo, 2010).

The average dietary energy intake per capita in Africa is 399 kcal for maize as food compared to a total intake of 80 kcal for Asia, 301 kcal for America, 59 kcal for Europe, and 38 kcal for Oceania. Within Sub‐Saharan Africa, the energy supply per day from maize is high in eastern and southern Africa (556.0 kcal/capita/day) compared to 287 kcal/capita/day for West and Central Africa and 318.3 kcal/capita/day for northern Africa (Erenstein et al., 2022). Despite these significant contributions of maize to human nutrition in Africa, the grain is highly susceptible to *Fusarium* infection and ZEA contamination, and their effect on health is extremely unnoticed in developing countries, including Africa.

## 3 RECENT ADVANCEMENTS IN THE IDENTIFICATION OF ZEARALENONE‐PRODUCING SPECIES

Improved new and emerging technologies are important for optimizing food safety tests. Several rapid tests have been developed and employed to detect *Fusarium* species and ZEA in food products. Among these methods, quantitative PCR (MitoqPCR, optimized qPCR, multiplex qPCR), loop‐mediated isothermal amplification (LAMP), PCR‐ELISA, metabarcoding, and microarray are frequently used. However, the identification of *Fusarium* species in Africa mainly depends on morphological growth parameters of the fungi and conventional PCR methods that are time‐consuming, laborious, and require specific expertise and experience (Table 1). Compared to morphological assays and singleplex PCR, multiplex PCR‐based technique has allowed the simultaneous detection of foodborne fungi belonging to distinct genera in a single reaction. This process mainly uses highly conserved and variable sequence regions such as β‐tubulin, elongation factor 1 α, and intergenic spacer region (IGS) of the rDNA that have demonstrated a high throughput and precise technology for characterizing *Fusarium* species. Although internal transcribed spacer (ITS) has been widely used in *Fusarium* species identification, it is now commonly accepted that internal transcribed spacer (ITS) is not informative enough to verify species identification within the *Fusarium* genus (Donnell et al., 2022; Summerell, 2019). Furthermore, conventional PCR assay is disadvantaged by its inability to quantify fungal load, high workload, high cost, and high turnaround time.

**TABLE 1:** Summary of recent *Fusarium* species occurrence in some African countries.

Country/region	Food crop	AT; time	Target DNA	Primers and probes (5′‐3′) or mode of identification	Identified species	OR	References
Eastern Africa							
Ethiopia	Maize	53°C; 50 s	EF‐1α	**EF1** ATGGGTAAGGAGGACAAGAC	*F*. *verticillioides*	42	Tsehaye et al. (2016)
				**EF2** GGAGGTACCAGTCATCATGTT	*F*. *graminearum*	22.6	
					*F*. *equiseti*	0.5	
Kenya	Wheat	57°C; 60 s	EF‐1α	**EF1** ATGGGTAAGGAAGGACAAGAC	*F*. *verticillioides*	30.0	Kheseli et al. (2021)
				**EF2** GGAGGTACCAGTCATCATGTT	*F*. *equiseti*	25.3	
	Maize	53°C; 30 s	TEF 1‐α	**EF1** GTGGGGCATTTACCCCGCC **EF2** ACGAACCCTTACCCACCTC	*F*. *verticillioides*	–	Alaro (2021)
Tanzania	Maize	62.5°C; 60 s		NS	*F*. *culmorum*	3.8	Degraeve et al. (2016)
					*F*. *graminearum*	75	
					*F*. *culmorum*	5	
					*F*. *sporotrichioides*	15	
					*F*. *verticillioides*	95	
Uganda	Maize, rice, millet	52°C; 30 s	EF‐1α	**rp32** ACAAGTGTCCTTGGGGTCCAGG **rp33** GATGCTCTTGGAAGTGGCCTACG	*F*. *verticillioides*	20	Wokorach et al. (2021)
	Sorghum		TEF 1‐α	**EF1** GTGGGGCATTTACCCCGCC **EF2** ACGAACCCTTACCCACCTC	*F*. *equiseti*	6.9	
					*F*. *proliferatum*	1.3	
Zambia	Maize	–	–	Morphological	*F*. *verticillioides*	37.1	Kankolongo et al. (2009)
Southern Africa							
South Africa	Commercial maize	58°C; 45 s	ITS	**ITS1** TCCGTAGGTGAACCTGCGG **ITS4** TCCTCCGCTTATTGATATGC	*F*. *oxysporum*	20	
					*F*. *verticillioides*	88	Chilaka et al. (2012)
					*F*. *proliferatum*	73	
					*F*. *oxysporum*	65	
					*F*. *graminearum*	48	
South Africa	Maize	58°C; 45 s	ITS	**FF2** GGTTCTATTTTGTTGGTTTCTA **FR1** CTCTCAATCTGTCAATCCTTATT	*F*. *verticillioides*	76	Ekwomadu et al. (2018)
					*F*. *oxysporum*	60	
					*F*. *graminearum*	18	
					*F*. *equiseti*	18	
					*F*. *solani*	1	
Angola	Maize	–	–	Morphological	*F*. *verticillioides*	83	Panzo (2011)
					*F*. *graminearum*	68	
					*F*. *graminearum*	22.5	
					*F*. *pseudoanthophilium*	13.4	
Botswana	Maize meal	–	–	Morphological	*F*. *verticillioides*	20.9	Mokgatlhe et al. (2011)
					*F*. *proliferatum*	2.5	
	Sorghum meal			Morphological	*F*. *verticillioides*	20	
					*F*. *proliferatum*	3.4	
Western Africa							
Burkina Faso	Onion	52°C; 30 s		**EF1** ATGGGTAAGGARGARGACAAGAC **EF2** GGARGTACCAGTSATCATCATGTT	*F*. *oxysporum* *F*. *proliferatum*	44.44 41.66	Kintega et al. (2020)
Nigeria	Oil palm	65°C; 30 s	ITS	**ITS1** TCTGTAGGTGAACCTGCGG **ITS4** TCCTCCGCTTATTGATATGC	*F*. *oxysporum* *F*. *equiseti* *F*. *verticillioides* *F*. *proliferatum*	41.37 20.68 5.17 3.44	Chidi et al. (2020)
Ivory Coast	Multiple samples	55°C; 45 s	ITS	**ITS1** TCCGTAGGTGAACCTGCGG **ITS4** TCCTCCGCTTATTGATATGC	*F*. *culmorum* *F*. *graminearum* *F*. *proliferatum*	7.4 18.5 25.9	Aasa et al. (2022)
Ghana	Groundnut	–	–	Morphological	*F*. *verticillioides*	20.7	Korley et al. (2022)
					*F*. *verticillioides*	31	
Nigeria	Maize	–	–	Morphological	*F*. *sporotrichioides*	96	Ezekiel et al. (2008)
					*F*. *verticillioides*	82	
					*F*. *graminearum*	50	
					*F*. *proliferatum*	40	
					*F*. *sporotrichioides*	76	
Northern Africa							
Morocco	Wheat	58°C; 1 min 55°C; 30 s	TEF 1‐α	**Fu3f** GGTATCGACAAGCGAACCAT **Fu3f** TAGTAGCGGGGAGTCTCGAA	*F*. *equiseti*	8.82	Ezrari et al. (2020)
				**FOF 1** ACATACCACTTGTTG CCTCG **FOR1** CGCCAATCAATTTGAGGAACG	*F*. *oxysporum*	17.65	
Egypt	Multiple products	55°C; 1 min	ITS	**ITS1:** TCTGTAGGTGAACCTGCGG **ITS4:** TCCTCCGCTTATTGATATGC	*F*. *equiseti* *F*. *verticillioides*		El‐Rabbat et al. (2018)
	Maize	58°C; 1 min	TEF	**F1T‐F** ATGGGTAAGGAGGACAAGAC **EF1T‐R** GGAAGTACCAGTGATCATGTT	*F*. *proliferatum* *F*. *culmorum* *F*. *oxysporum*	19.0 52.3 14.3	Khalil et al. (2013)
Algeria	Wheat	60°C; 30 s		**Fco1F** ATGGTGAACTCGTCGTGGC **Fco1R** CCCTTCTTACGCCAATCTCG	*F*. *culmorum*	68	Abdallah‐Nekache et al. (2019)
		55°C; 30 s		**Fp1 1**CGGGGTAGTTTCACATTTCYG **Fp1 2** GAGAATGTGATGASGACAATA	*F*. *pseudograminearum*	10	
		57°C; 30 s		**Fum 1–654** CGGTTGTTCATCATCTCTGA **Fum 1–654** GCTCCCGATGTAGAGCTTGTT	*F*. *verticillioides*	3	
Algeria	Wheat	62°C; 5 s		**Fcu‐F** GACTATCATTATGCTTGCGAGA**G** **Fcu‐R** CTCTCATATAC	*F*. *culmorum*	40	Hadjout et al. (2022)
Tunisia/Egypt	Sorghum	60°C; 53°C	β‐tubulin, and TEF‐1a	**BT2A** GGTAACCAAATCGGTGCTGCTTTC **BT2B** ACCCTCAGTGTAGTGACCCTTGGC **ET1:** ATGGGTAAGGARGACAAGAC **EF2:** GGARGTACCAGTSATCATGTT	*F*. *equiseta complex* *F*. *verticillioides* *F*. *proliferatum* *F*. *graminearum*	62.7 6.7 3.4 1.7	Lahouar et al. (2015)
Central Africa							
Cameroon	Maize, peanut, poultry feed	55°C; 20 s	IGS region	**IGSF** AAGGAATTCAGGAATTCTCAATTG **IGSR** GTCCACCGGCAAATCGCCGTGCG	*F*. *verticillioides*	90	Kana et al. (2013)

Abbreviation: OR, occurrence rate.

### 3.1 Quantitative PCR (qPCR)

Quantitative PCR (qPCR) is a variant of PCR technology that allows the real‐time detection of amplicons as they accumulate. It is important to acknowledge that the widespread adoption of qPCR is not without its challenges. One of the well‐known issues with quantitative PCR is its inability to discriminate between viable and nonviable *Fusarium* cells. This mostly results in the overestimation of viable *Fusarium* cells and the overuse of chemicals. To address this limitation, Chen et al. (2022) developed an improved method for quantifying viable *Fusarium* cells using propidium monoazide (50 mmol/L optimum) coupled with real‐time PCR. This method was able to reliably differentiate between viable *Fusarium* spores from nonviable spores. Furthermore, the qPCR setup is that each target gene needs its own dye and associated probe with a unique wavelength. Furthermore, qPCR results depend on a calibration curve, involving several calculations, and may not represent the number of *Fusarium* copies in the sample (Cao et al., 2020). Complex biological molecules such as humic acid significantly inhibit qPCR reactions.

### 3.2 Digital PCR (dPCR)

Digital PCR (dPCR) is a powerful tool for quantifying the absolute copy number of target DNA or RNA. It plays a crucial role in measuring genetic imbalances that result from an uneven distribution of targets, particularly in mycotoxin analysis. Among the various types of dPCR partitioning methods that have been explored for food safety management, microfluidic chip, and droplets, digital PCR have been examined in fungal biology and mycotoxin detection. With the droplet dPCR system, each DNA sample is mixed with oil, forming a water–oil emulsion. The water–oil emulsion is then divided into many thousands of single nanoliter droplets. During PCR, targets are amplified within each droplet. Recently, a ddPCR assay was developed by Wang et al. (2021) for the simultaneous quantification of 3‐acetyl deoxynivalenol (3ADON) and 15‐acetyl deoxynivalenol (15ADON) chemotypes of DON‐producing *Fusarium* species. Furthermore, chip digital PCR (cdPCR) was found to accurately track mycotoxigenic *Fusarium* DNA in wheat and oat plants during the initial phase of infection when symptoms are not visible (Morcia et al., 2020). The technology has not been used in zearalenone detection and quantification. Droplet digital polymerase chain reaction (ddPCR) has demonstrated its power in detecting gene targets differing in one nucleotide. Through not *Fusarium*, ddPCR was found to accurately distinguish the competitiveness between nonaflatoxigenic and aflatoxigenic *Aspergillus flavus* strains on maize kernels (Schamann et al., 2022).

### 3.3 Polymerase chain reaction–enzyme‐linked immunosorbent assay (PCR‐ELISA)

Polymerase chain reaction–enzyme‐linked immunosorbent assay (PCR‐ELISA) is a nucleic acid amplification assay that combines immunodetection method (ELISA) and PCR to quantify specific PCR product directly after immobilization of DNA on a microtiter plate. Compared to qPCR, PCR‐ELISA is sensitive and specific, uses less time, simultaneously detects a large number of samples, and involves fewer carcinogenic chemicals (Tayebeh et al., 2017). Grimm and Geisen (1998) proposed and developed a PCR‐ELISA for the detection of potential *Fusarium* species using the ribosomal ITS1 sequence as a target. Subsequently, Omori et al. (2018) explored the possibility of using PCR‐ELISA to detect *Fusarium verticillioides* in corn. The authors targeted the FUM21 gene for *Fusarium verticillioides* and confirmed that the technique was specific to *Fusarium verticillioides* isolates, and exhibited 100‐fold more sensitivity than the conventional PCR assay. However, higher DNA concentration (>250 pg) decreases the sensitivity of the detection method (Omori et al., 2018).

### 3.4 Loop‐mediated isothermal amplification

Loop‐mediated isothermal amplification is a novel amplification for faster detection of nucleic acids. Compared to PCR assays that require three different temperatures (initial temperature, annealing temperature, and the final extension temperature), LAMP technology utilizes a single temperature (Isothermal) and does not require a thermocycler. More recently, few studies have accessed the potential of using LAMP assay to determine *Fusarium* species and mycotoxin in maize. Wigmann et al. (2020) developed a direct LAMP‐based assay for detecting *Fusarium* species and mycotoxins in ungrounded maize grains. In their methodology, 1 g of maize grains was soaked with 2.5 mL of sterile tap water containing 1% (v/v) Tween 20. After handshaking, 2 mL of the supernatants was transferred into a reaction vessel and centrifuged at 11,000 *g* for 2 min. Subsequently, the Tween 20 was removed by washing with sterile deionized water and the pellet was resuspended in 500 μL of sterile deionized water. Five microliters of the suspension was used as a template in LAMP reactions. This method was able to detect *Fusarium verticillioides* with the potential to produce fumonisin even at lower concentrations.

### 3.5 Full genome sequencing

Although the full genome of several ZEA‐producing *Fusarium* isolates from different parts of the world has been sequenced and used in the bioinformatic analysis, there are no similar studies of isolates originating from Africa. The first *Fusarium* genome to be sequenced completely in Africa was the South Africa's *Fusarium circinatum* type strain FSP34 (Wingfield et al., 2012). This strain was sequenced on a Roche 454 GS FLX system (Life Sciences, Connecticut, USA) using titanium chemistry and it was found that the strain contains about 44 million bases in size, 15,000 open reading frames with putative gen clusters harboring evidence of the secondary metabolites of fumonisin and fusarin. About 70% of the *Fusarium circinatum* open read frames were most similar to those of *Fusarium verticillioides* (Wingfield et al., 2012).

Compared to the current partially sequenced genomes widely used to study *Fusarium* isolate in Africa, complete genomes provide detailed bioinformatic information including the size, heterozygosity, repetitive sequence content, and interspecific and intraspecific comparison, and facilitate all aspects of biological research. Complete genome sequencing has also shed light on the evolution of the pathogenicity history of various *Fusarium* fungi and allows the selection of several primer pairs used in PCR reactions for molecular characterization of the specific mycotoxigenic chemotypes. To this end, different primer sets have been designed for rapid and specific detection of ZEA‐producing *Fusarium* species in contaminated foodstuff such as maize flour. Based on the gene sequence of polyketide synthase PKS4, Meng et al. (2010) developed a low‐cost technique using a specific primer set that was rapid, sensitive, specific, and reliable in the identification and quantification of ZEA‐producing *Fusarium* species directly in maize flour. Similarly, Atoui et al. (2011) developed a real‐time PCR assay from a polyketide synthase gene PKS13 sequences involved in ZEA biosynthesis and successfully detected and quantified *Fusarium graminearum* and was used to analyze the occurrence of 32 ZEA‐producing *F*. *graminearum* chemotypes on maize.

## 4 GENES EXPRESSION IN ZEA PRODUCTION

Molecular techniques such as differential display RT‐PCR (DDRT‐PCR) and expressed sequence tags approaches have been used to understand the molecular mechanisms connected with ZEA production in maize genotypes. Two polyketide synthase genes PKS4 and PKS13, transcription factor (ZEB2), and genes similar to isoamyl alcohol oxidase (ZEB1) as well as other regulatory proteins have been identified and reported as genes expressed in ZEA production (Lysøe et al., 2006). Lysøe et al. (2008) used differential display RT‐PCR to identify 54 expressed sequence tags that were upregulated in rice samples with higher ZEA production. Furthermore, the MIPS Functional Catalogue (FunCat) of classifying expressed genes classified these upregulated genes into five major functional categories: 38% unclassified proteins, 15% metabolism, 10% proteins with binding function, 6.5% biogenesis of cellular components, and 5.5% cell rescue, defense, and virulence (Lysøe et al., 2008). Using microarray analysis on *F. graminearum* strains.

Lee et al. (2011) found significantly elevated ZRA1 gene levels, a putative ATP‐binding cassette transporter gene, in test samples containing 15 μM ZEA compared with those without ZEA supplements. It has been suggested that these genes are expressed between 4 and 14 days after infection with the necessary growth conditions (Lysøe et al., 2006, 2008). This particular finding is significant that plant debris can transfer ZEA and its conjugate metabolites into the soil and subsequently translocate ZEA to other plant parts including leaves, stems, grains, and fruits (Gerling et al., 2022; Jaster et al., 2023; Rolli et al., 2018).

## 5 ZEARALENONE AND ITS PRODUCING FUNGI IN SUB‐SAHARAN AFRICA

Zearalenone, a metabolite produced by various species of *Fusarium* fungal, has been observed as a natural contaminant of cereals, in particular maize, in many countries in Africa, Europe, and the USA. Globally, high levels of ZEA production are associated with *Fusarium graminearum* and *Fusarium culmorum*. However, *F*. *oxysporum*, *F*. *sporotrichioides*, *F*. *proliferatum*, *and F*. *verticillioides* have also been linked to ZEA production (Beev et al., 2013). Although these fungi have been widely reported to colonize a variety of food crops including maize, sorghum, wheat, rice, millet, and other legumes products, recent evidence suggests that ZEA can also contaminate water, meat, fish, and dairy commodities (Alaboudi et al., 2022; Falkauskas et al., 2022; Gonkowski et al., 2018; Jafari‐Nodoushan, 2022).

**FIGURE 1:**
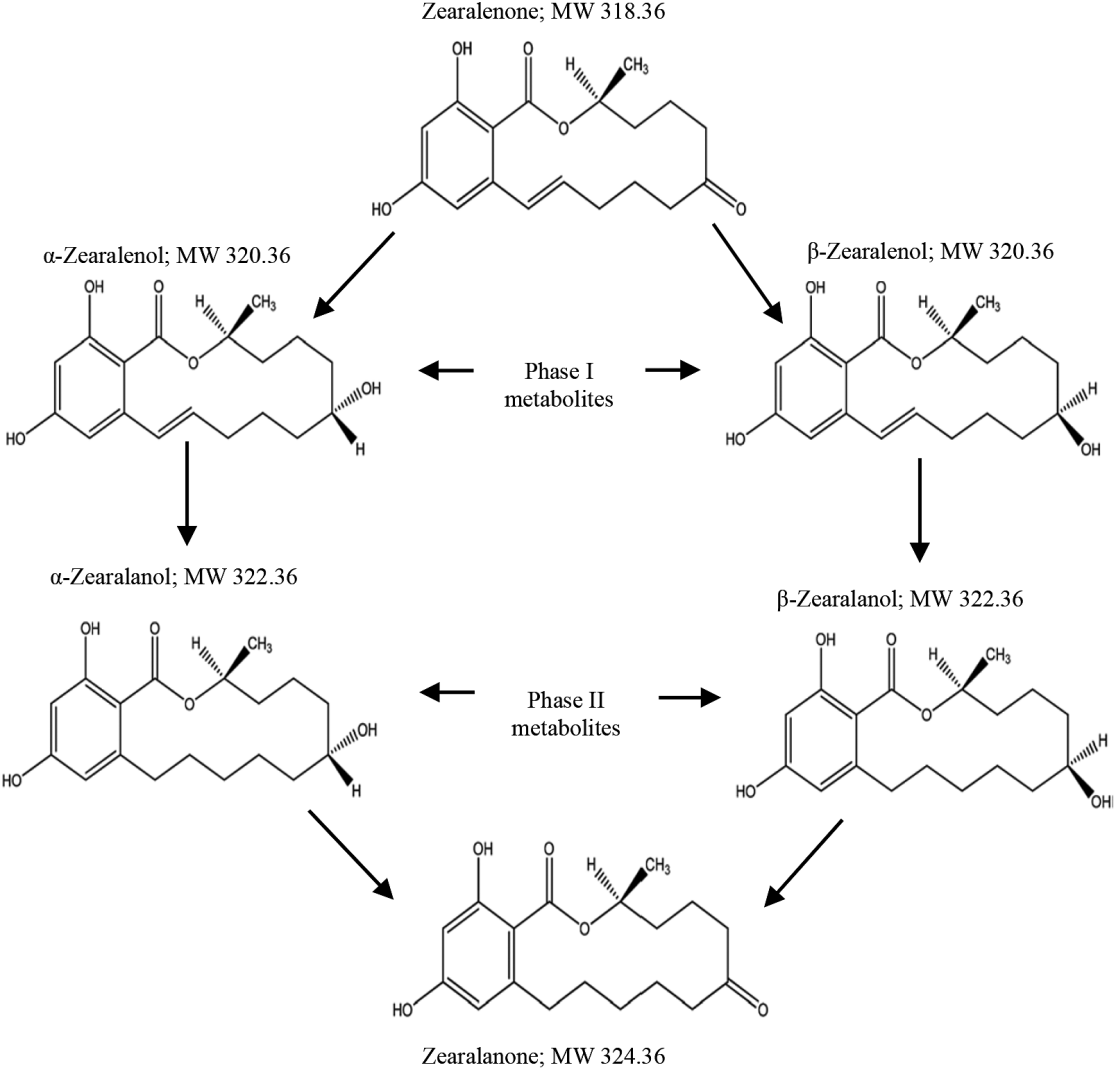
Chemical structure of zearalenone and its associated metabolites.

The chemical structure of ZEA and its five known conjugate metabolites are shown in Figure 1. The toxin is soluble in solvents such as acetonitrile, acetone, methyl chloride, and alcohol. In terms of stability, ZEA is stable to conventional food processing temperatures (EFSA, 2011). Globally, ZEA and its metabolites are well known for their estrogenic activities, resulting in reproductive system dysfunction. Furthermore, ZEA exhibits cytotoxicity by modifying biological macromolecules such as DNA, proteins, and nucleic acid (Eze et al., 2018; Jafari‐Nodoushan, 2022; Rai et al., 2020).

Over the past decade, morphological and molecular analysis performed on various food crops showed the presence of several fungi genera. Among these fungi genera, *Fusarium* species are the most dominant fungi frequently isolated from grains in Africa (Akello et al., 2021; Ekwomadu et al., 2018; John et al., 2018). The most common isolated *Fusarium* species from food crops in Africa are *F*. *graminearum*, *F*. *verticillioides*, *F*. *culmorum*, *F*. *sporotrichioides*, and *F*. *equiseti* (Table 2). Their occurrence rate ranged from 3% to 100%, showing the ubiquitous presence of potential mycotoxigenic *Fusarium* species in Africa. These species have been isolated from several grains, including maize, wheat, and sorghum. Table 1 provides a summary of published accounts on the *Fusarium* species prevalence rate in Africa as well as their detection methods.

**TABLE 2:** Impact of oxidative stress factors on *Fusarium* species activities.

Stress compound	Effect on *Fusarium* growth, and mycotoxin production	*Fusarium* strain used	References
Sodium dodecyl sulfate (0.02%)	Decrease FUM gene expression	*F*. *verticillioides*	Nagygyörgy et al. (2014)
H_2_O_2_ (0.5, 2.0 mM)	Increase fumonisin production by up to 300% Enhance FUM gene expression	*F*. *verticillioides*	Ferrigo et al. (2015)
H_2_O_2_	Increase DON and acetyl‐DON production Enhance TRI gene expression	*F*. *graminearum*	Ponts et al. (2007, 2009)
H_2_O_2_ (0.05%)	Reduce fungi growth rate	*F*. *graminearum*	Zheng et al. (2012)
SDS (0.05%)	Enhance the growth of colony diameter	*F*. *graminearum*	Zheng et al. (2012)
H_2_O_2_ (0.5 mM)	Increase DON and NIV production	*F*. *culmorum*	Ponts et al. (2009)
NaCl	Increased mycelial growth, mycelial biomass, sporulation, and microconidia	*F*. *oxysporum*	Maharshi et al. (2021)
EC (2–4 dS/m)	Significantly enhanced fungal growth Increased biomass of fungal up to 90%	*F*. *oxysporum*	Shoaib et al. (2018)
Sublethal dose Prothioconazole	Increase DON production	*F*. *graminearum*	

## 6 LEGISLATION ON ZEARALENONE IN AFRICA

As part of managing the exposure risk of animals and humans to different toxic compounds, some countries, including the European Union (European Commission, 2006a, 2006b), Brazil (Corrêa & Ferreira, 2023), and Asia/Oceania (FAO, 2004), have established regulatory limits for mycotoxin concentrations in food and feedstuff. Currently, the UDA has not established regulatory levels for ZEA contamination in grain or food products. The WHO/FAO set 200 g/kg bw/day for pigs and gilts, 17.6 g/kg bw/day for piglets, 56 g/kg bw/day for sheep, and 20 g/kg bw/day for dogs as the lowest observed adverse effect level (LOAEL) for ZEA.

For many African countries, most of these regulations are limited to aflatoxins and fumonisin (Chilaka et al., 2022). Data on ZEA occurrence and evidence of high dietary exposure in individual countries are limited for use in the establishment of maximum permitted levels in food and feed raw material. As a result, many of these countries have adopted the European Commission and Codex Alimentarius Commission standards for ZEA (Ankwasa et al., 2021; Imade et al., 2021; Lahouar et al., 2018). However, only South Africa and Morocco have set regulatory limits for ZEA concentration in food and feed products. In South Africa, the maximum limits for ZEA in feeding stuff for sow/pigs, piglets, and calves/dairy cattle have been pegged at 5000, 3000 μg/kg, and 500 μg/kg, respectively (Government Notice, 2010). In Morocco, Zinedine and Mañes (2009) proposed ZEA limits of 200 μg/kg for all cereals intended for human consumption.

## 7 CONTRIBUTION OF PREHARVEST AND POSTHARVEST FACTORS TO *FUSARIUM* INFESTATION AND ZEARALENONE ACCUMULATION IN FOOD

**FIGURE 2:**
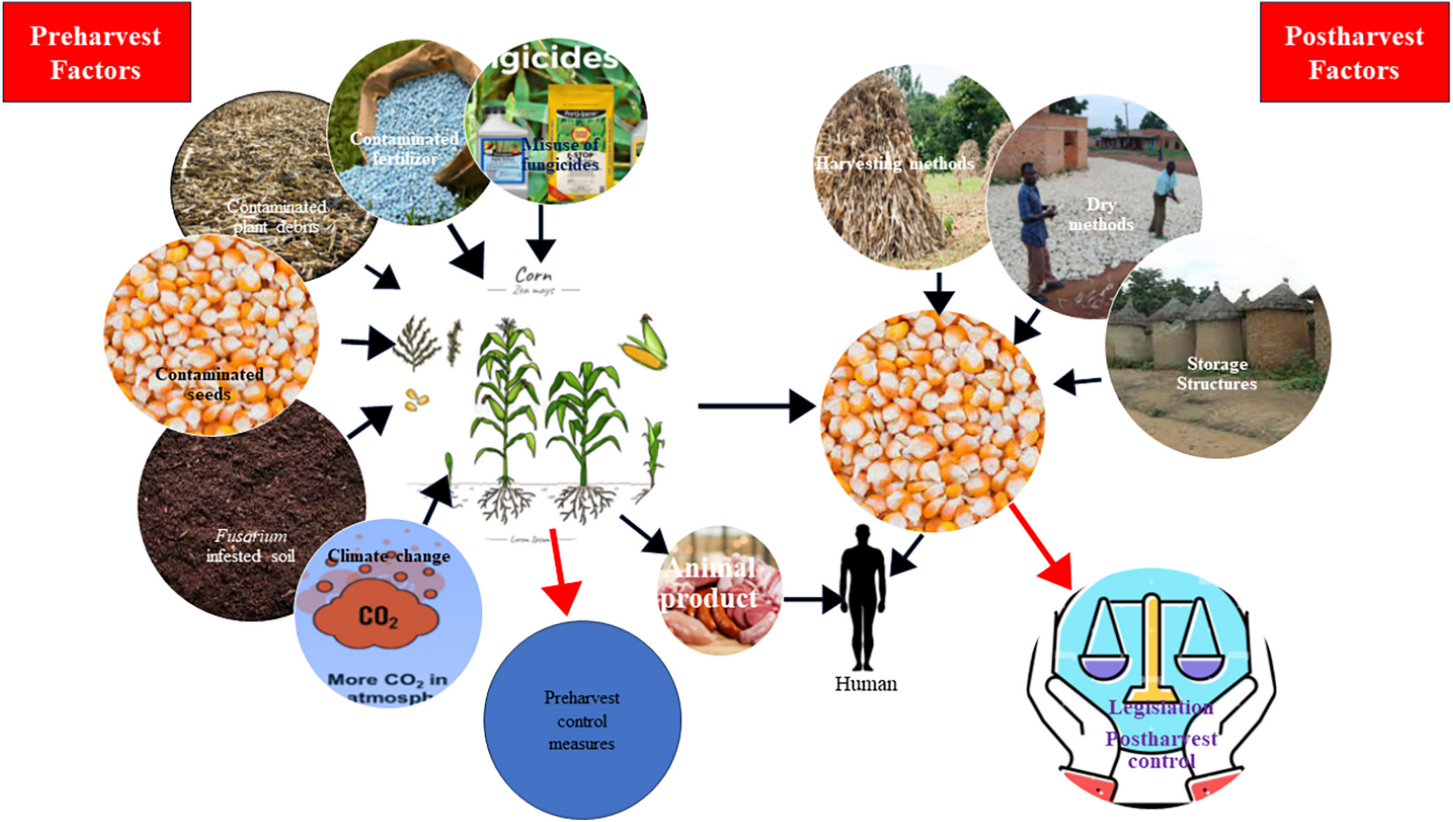
Maize grain and human exposure routes to ZEA contamination in food.

Preharvest practices high in repeated misuse of chemical fertilizer and farmer‐saved seeds, poor harvesting conditions, poor drying conditions, and poor storage structures result in fungi growth and mycotoxin production (Lehmane et al., 2022; Tang et al., 2019). Hence, ZEA concentrations in food are underpinned by three powerful factors—Preharvest factors identified as fertilization levels, fungicides use, and harvest practices; postharvest factors such as drying techniques, storage conditions; and food processing factors including sorting and fermentation. Figure 2 provides a summary of maize exposure routes to *Fusarium* infestation and ZEA contamination.

### 7.1 Nitrogen fertilization

It is well established that nitrogen fertilizer increases maize susceptibility to *Fusarium* attack and ZEA accumulation through increased oxidative stresses (Borràs‐Vallverdú et al., 2022). The type of stress significantly impacts the kind of mycotoxins observed in maize grains. It has become something of a truism that high nitrogen doses (>200 kg N/ha) increased ZEA accumulation in maize grains (Blandino et al., 2008; Podolska et al., 2017; Scarpino et al., 2022). However, the type of nitrogen fertilizer did not have a significant impact on ZEA accumulation in maize grains (Blandino et al., 2008).

### 7.2 Use of fungicides

The use of fungicides has been extensively reported in Africa; however, often of over or under (sublethal) applications, a phenomenon that may trigger the production of various mycotoxins. This is so because fungicides have the potential to induce the production of hydrogen peroxide (H_2_O_2_), which is a key precursor that influences the biosynthesis pathway of secondary metabolites (Audenaert et al., 2010; Ferrochio et al., 2013). The impact of fungicides on the biosynthesis of mycotoxins using *Fusarium* species has been reported. Cendoya et al. (2021) studied the impact of commercial fungicides: epoxiconazole + metconazole, tebuconazole, pyra‐clostrobin + epoxiconazole, and prothioconazole on fumonisin accumulation and observed that all the studied fungicides used in sublethal doses enhanced fumonisin production except prothioconazole. The impact of oxidative stress on *Fusarium* mycotoxins is summarized in Table 2. However, the influence of oxidative stress on ZEA accumulation and ZEA gene expression has not been reported globally.

### 7.3 Harvesting operations

In Ethiopia and most countries in Africa, farmers use dry and bent‐down kernels that are completely dry and show a black layer at the base to determine harvest time (Mohammed, Bekeko, et al., 2022; Mohammed, Seid, et al., 2022). Other indigenous methods used by farmers to estimate maize moisture content for harvesting and storage are puncturing kernels with their thumbnails, biting kernels with their teeth, or using sound made by kernels when agitated by hand (Joseph et al., 2015; Kagot et al., 2022; Liu et al., 2016). Such harvesting operations may influence *Fusarium* infestation and ZEA production. A study conducted to evaluate the impact of grain maturation, harvesting time, and late‐season rainfall showed that grains harvested late and exposed to preharvest rainfall had higher ZEA than those harvested early and not subjected to rainfall (Moraes et al., 2022). Recent data indicate that for every 1‐day delay in harvesting, ZEA production is projected to increase by 0.8‐fold in grains (Edwards & Jennings, 2018). It was previously reported that hay dried on the field had more ZEA than those dried under the shed (Taffarel et al., 2013).

### 7.4 Storage conditions

In Africa, storage methods and structures differ by ethnic groups and agroecological area. Most storage structures in Africa are characterized by high relative humidity and temperatures. These structures and environmental conditions result in high relative humidity (>60%), temperature buildup, and insect propagation which may lead to ZEA accumulation   (Tang et al., 2019). A study conducted in three climatic locations in Africa on the influence of storage practices on ZEA contamination in rice showed that storage location (N'diaye in Senegal, Cotonou in Benin, and Yaoundé in Cameroon), processing type (plastic woven bags and pallets), and duration of storage (0, 90, and 180 days) affect ZEA concentration. The authors observed that storage structures with high relative humidity and low temperatures recorded significantly increased levels of ZEA (80% RH; 24.4°C; 400.3 ppm ZEA) for grains stored for 6 months compared to storage structures with low relative humidity (62.8% RH; 27.8°C; 100.2 ppm ZEA).

Similarly, temperature variations affect ZEA levels in grains under storage. High levels of ZEA (51.8–468.6 ppm) were observed in grain spikes exposed to 100% relative humidity at different temperatures. At relative humidity ≤90%, ZEA concentrations were very low (0.1–3.6 ppm) at all tested temperatures. At 100% relative humidity, mean ZEA contamination with significantly higher at 20 and 25°C (235.1 and 278.2 ppm) than at 30°C (104.7 ppm) (Moraes et al., 2022). Additionally, improper cleaning of grains before storage may lead to *Fusarium* and ZEA contamination of grains. High proportions of plant debris such as cobs and husks may increase ZEA levels in the stored grains due to contaminated plant debris in grains (Bamba et al., 2020; Tang et al., 2019).

### 7.5 Transboundary trade and zearalenone occurrence

Although reports from Africa are not consistent with levels of ZEA detected in imported and locally produced products, transboundary trade has emerged as one major factor contributing to the spread and distribution of mycotoxigenic fungi. A survey carried out in Nigeria has reported higher concentration levels of deoxynivalenol, and ZEA in imported wheat grains (mean 858.7 μg/kg for deoxynivalenol; 50.1 μg/kg for ZEA), whereas relatively lower levels (mean 517.8 μg/kg for deoxynivalenol; 14.5 μg/kg for ZEA) were detected in samples obtained from local farmers' stores or markets (Egbontan et al., 2017). In a similar study, Manizan et al. (2018) evaluated the occurrence of ZEA in local and imported grains in Cote D'Ivoire and did not detect ZEA in the 41 imported rice samples; however, 44.4% of the local samples being contaminated with ZEA at varying levels.

## 8 IMPACT OF CLIMATE CHANGE ON ZEARALENONE AND *FUSARIUM* OCCURRENCE

Climate change has already carried along an increased risk of natural toxins through disruption of plant metabolic and cellular processes. Among these climate variables, elevated atmospheric CO_2_ concentration, temperature, and precipitation are predicted to affect mycotoxigenic fungi redistribution and their derived mycotoxins (Medina et al., 2017). Recent reports suggest that elevated CO_2_ had a significant correlation (*R* ^2^ = .938; *p* < .001) with ZEA accumulation (Cui et al., 2022). Atmospheric CO_2_ concentration has also been shown to influence disease severity caused by *Fusarium* species. Likewise, higher emission of CO_2_ increases maize susceptibility to *F*. *verticillioides* attack (Vaughan et al., 2014), *F*. *culmorum* (Bencze et al., 2017), and *F*. *graminearum* (Hay et al., 2021).

Recent projected global climate status showed that Africa will experience extreme precipitation and temperature events like heat waves and drought. Extreme environmental temperatures combined with high precipitation amounts or prolonged droughts increase the level of stress suffered by plants, making all cereals, especially maize, even more, prone to fungi infection and mycotoxin contamination (Kos et al., 2023). For *Fusarium* mycotoxins, high ZEA concentrations are observed particularly at the flowering and harvesting stage, with extreme levels of precipitations (Han et al., 2022; Janić Hajnal et al., 2023). It is worth mentioning that *Fusarium* strains isolated from Africa are more resilient to extreme temperatures and lower water potential (Jedidi et al., 2021).

## 9 INCIDENCES AND LEVELS OF ZEARALENONE CONTAMINATION IN AFRICA

### 9.1 Zearalenone in cereal and cereal products

Zearalenone is a stable mycotoxin and is not degraded under storage (Krska et al., 2003). Several global surveys found a higher incidence rate of ZEA in Africa, with varying incidence rates and levels among grains. A recent global survey showed a high prevalence rate of ZEA in food and feed samples; for example, a global survey conducted from 2008 to 2017 showed a higher prevalence rate of ZEA prevalence in Sub‐Saharan Africa (52.2%) and South Africa (41.6%) than in Europe, America, and South Asia (Gruber‐Dorninger et al., 2019). Similarly, Lee and Ryu (2017) showed higher incidences of ZEA in unprocessed cereals from Africa (59%) than in Europe and America (48%).

In eastern Africa, Mohammed et al. (2023) profiled multiple mycotoxins in 80 postharvest maize grains from three major producing areas in Ethiopia: of the mycotoxins analyzed, ZEA was detected in 81%, with levels as high as 3750 μg/kg. Previously in Ethiopia, ZEA concentrations were investigated in a total of 100 maize samples collected from smallholder farmers' stores (Getachew et al., 2018). ZEA was detected in 96% of the samples, with mean and maximum concentrations of 92 and 1656 μg/kg, respectively. Of the 96% positive samples, 13.5% contained levels above the European Union (EU) recommended value for unprocessed cereals (100 μg/kg). In another study in south and south‐western Ethiopia, Mesfin et al. (2021) randomly sampled 176 stored maize from various households and analyzed them for *Fusarium* mycotoxins: ZEA concentration was up to 2447 μg/kg. In Tanzania, Kamala et al. (2015) investigated multiple mycotoxin levels in 300 maize samples collected from various rural households. For the ZEA‐positive samples (10%), 66% contained levels exceeding the EU maximum permitted limits. In a later study by Suleiman et al. (2017), 30 samples of maize purchased from farmers and traders in Tanzania were analyzed for ZEA. All 30 samples tested positive for ZEA with levels ranging from 50 to 189.9 μg/kg. More recently in Kenya, maize samples collected between 2018 and 2020 for multiple mycotoxin profiling revealed that 18% of the 480 maize samples collected from Kenyan households had ZEA levels above 1000 μg/kg (Kagot et al., 2022).

Several studies in southern Africa have also reported maize contamination with ZEA. In a survey conducted between 2001 and 2021 in Eswatini, 892 maize and maize‐based products were sampled and screened for ZEA contamination. In these samples, 7% of maize grain and 17% of maize meal were contaminated with ZEA (Dlamini et al., 2022). A total of 100 maize samples were randomly selected from small‐scale and commercial farmers in South Africa and investigated for ZEA contamination using HPLC. The results showed that more than half of the small‐scale and commercial maize samples were contaminated with ZEA at mean levels below the South African regulatory limits (Ekwomadu et al., 2021). In Zimbabwe, a study conducted to assess the impact of storage duration (0, 90, and 180 days) on ZEA contamination in maize showed an increase in ZEA concentration by 89.6% and 173.6% in 90 and 180 days storage periods, respectively (Hove et al., 2016).

Within the western Africa subregion, higher concentration levels of ZEA have been observed in recent studies. For instance, in Côte d'Ivoire, 125 samples of maize were analyzed for ZEA, and all the samples were contaminated with ZEA with 40% of the samples exceeding the EU regulatory limits (Bamba et al., 2020). In 2019 and 2021, Oyeka et al. (2019) and Olopade et al. (2021), respectively, investigated ZEA concentrations in maize from different agroecological zones in Nigeria. While none of the samples analyzed by Olopade et al. (2021) had levels exceeding the maximum permitted levels, 16.7% of the 36 maize samples analyzed by Oyeka et al. (2019) had ZEA concentrations above the EU maximum level for maize intended for direct human consumption. Additionally, all 20 samples were positive for ZEA phase I metabolites; alpha and beta zearalenol (Olopade et al., 2021). In another study in Togo, ZEA was detected in only one of 52 maize samples in lower concentrations (Hanvi et al., 2019).

Generally, the incidence rate of ZEA is fairly low for maize samples from northern Africa. Mahdjoubi et al. (2020) evaluated Algerian maize kernels from different local markets and reported that only one of seven ZEA‐positive samples exceeded the EU maximum permitted levels. In Egypt, Sebaei et al. (2020) evaluated multiple cereals including 55 maize samples for ZEA contamination and observed that two samples had ZEA at levels 10 and 108 μg/kg. Previous research by Abdallah et al. (2017) also revealed that 10 out of 79 maize samples collected from farms and market centers in Egypt were contaminated with ZEA.

Meanwhile, data on ZEA occurrence in maize grains from Central Africa are quite real. A survey conducted on multiple products including 37 maize samples in Cameroon revealed that about 89% of the maize samples were contaminated with ZEA in levels ranging from 0.2 to 309 μg/kg (Abia et al., 2013). In the Democratic Republic of Congo (Mulunda et al., 2013), 40 maize samples collected from main markets were analyzed for various *Fusarium* mycotoxins. For ZEA, 92.5% of the samples were positive with concentrations ranging from 24 to 811.2 μg/kg.

### 9.2 Zearalenone in processed food and beverages

A survey evaluated ZEA levels in 50 traditionally maize fufu collected from households in Bamunka in Cameroon. Forty percent and 90% of the samples contained detectable levels of alpha and beta zearalenol. All 50 traditional maize fufu samples were ZEA positive with levels ranging from 5 to 150 μg/kg (Abia et al., 2017). In another survey, 101 maize porridge samples from households across three rural villages in Tanzania were evaluated for mycotoxins. ZEA was detected in 31% of the samples with levels ranging from 10.20 to 269.9 μg/kg (Geary et al., 2016). In another study in South Africa, Shephard et al. (2013) analyzed maize‐based evening meals and porridge donated by 54 females in Transkei, a region with high esophageal cancer for multiple mycotoxin occurrence. For ZEA, the author observed that all the samples were contaminated with ZEA with values ranging from 0.2 to 239 μg/kg for maize‐based food and 0.44 to 239 μg/kg for porridge samples. Fast forward to Limpopo (South Africa), analyses of 20 maize porridge showed no contamination of ZEA and beta zearalenol. However, alpha zearalenol was detected in 19 of the 20 samples at levels between 10.25 and 61.5 μg/kg (Tebele et al., 2020). A study on the occurrence of ZEA in cooked maize porridge and dietary exposure of Tanzanian households to ZEA was performed in 2016. It was reported that 23% of the processed maize‐based porridge had ZEA concentration above the limit for infant food (ZEA > 20 μg/kg) (Geary et al., 2016).

In Cameroon, a selection of 14 traditional maize beers and eight dagwa—a dry fried snack made from milled maize and groundnuts—samples produced from maize were screened for the presence of ZEA and other mycotoxins. Of these samples, 86% of the traditional beer and 100% of the dagwa samples were confirmed to contain ZEA in levels ranging from 1.6 to 35 μg/kg and 6 to 57 μg/kg, respectively (Abia et al., 2013). The authors further observed that mycotoxin‐related knowledge was low among fermented food sellers. Contrary to this observation, Adekoya, Njobeh, et al. (2017) and Adekoya, Obadina, et al. (2017) evaluated 32 maize‐based beers from South Africa for the cooccurrence of mycotoxins and reported that none of the samples contained detectable levels of ZEA despite the detection of fumonisin in 53% of the samples. Tables 3 and 4 provide a summary of published accounts on ZEA levels in other food products and beverage samples in Africa.

**TABLE 3:** Summary of zearalenone occurrence in raw food and feed from Africa.

Country/region	Food crop	Sample size (positive sample)	Detection method	Mean (range) (μg/kg)	References
Eastern Africa					
Kenya	Fish feed	78 (40)	HPLC	136 (<38–757.9)	Mwihia et al. (2020)
Ethiopia	Sorghum	80 (19)	HPLC	3.62 (<LOD‐121)	Mohammed, Bekeko, et al. (2022) and Mohammed, Seid, et al. (2022)
Kenya	Animal feed	25 (19)	HPLC	67 (61–167)	Rodrigues et al. (2011)
Kenya	Feed	10 (6)	LC–MS/MS	NS (11.2–28.2)	Warth et al. (2012)
Tanzania	Cassava	405 (154)	LC–MS/MS	NS (21.4–8493)	Sulyok et al. (2015)
Rwanda	Cassava	222 (104)	LC–MS/MS	NS (100–2826)	Sulyok et al. (2015)
Southern Africa					
South Africa	Chicken feed	62 (62)	LC–MS/MS	100 (NS‐610)	Njobeh et al. (2012)
South Africa	Cattle feed	25 (24)	LC–MS/MS	72 (NS‐123)	Njobeh et al. (2012)
South Africa	Horse feed	3 (3)	LC–MS/MS	43 (NS‐46)	Njobeh et al. (2012)
South Africa	Swine feed	2 (2)	LC–MS/MS	148 (NS‐170)	Njobeh et al. (2012)
Madagascar	Cassava	126 (41)	LC–MS/MS	83.8 (16.3–286.7)	Abass et al. (2019)
Namibia	*Kalaharituber pfeilii*	8 (8)	ELISA	NS (45–9680)	Hainghumbi et al. (2022)
Western Africa					
Côte d'Ivoire	Cobs	125 (125)	HPLC	NS (38.61–234.87)	Bamba et al. (2020)
Côte d'Ivoire	Spathes	125 (125)	HPLC	NS (94.54–341.84)	Bamba et al. (2020)
Nigeria	Sorghum	20 (18)	LC–MS/MS	18 (<LOQ‐20)	Olopade et al. (2021)
Nigeria	Millet	20 (17)	LC–MS/MS	64 (<LOQ‐396)	Olopade et al. (2021)
Nigeria	Granola	18 (11)	LC–MS/MS	1.73 (0.81–5.99)	Ezekiel et al. (2020)
Nigeria	Popcorn	19 (1)	LC–MS/MS	6.40	Ezekiel et al. (2020)
Nigeria	Millet	87 (14)	LC/MS	419 (0–1399)	Chilaka et al. (2016)
Nigeria	Rice	41 (33)	HPLC	203.6 (0.7–570.6)	Egbuta et al. (2015)
Togo	Sorghum	12 (2)	LC–MS/MS	22 (19–24.6)	Hanvi et al. (2019)
Northern Africa					
Tunisia	Wheat	155 (123)	HPLC	110 (0–560)	Zaied et al. (2012)
Algeria	Wheat	30 (19)	UHPLC–MS/MS	102 (9.6–295)	Mahdjoubi et al. (2020)
Algeria	Rice	30 (6)	UHPLC–MS/MS	9.9 (8.6–15.5)	Mahdjoubi et al. (2020)
Egypt	Wheat	15 (6)	HPLC	1.55 (0.53–2.5)	El‐Desouky and Naguib (2013)
Egypt	Barley	15 (4)		1.5 (0.70–1.77)	El‐Desouky and Naguib (2013)
Egypt	Animal feed	77 (71)	LC–MS/MS	NS (NS‐791)	Abdallah et al. (2017)
Central Africa					
Cameroon	Edible nontimber products	210 (194)	ELISA	62.7 (<15–500)	Djeugap et al. (2019)
Cameroon	Peanut	35 (15)	LC–MS/MS	4 (<LOQ‐45)	Abia et al. (2013)
Cameroon	Soybean	10 (10)	LC–MS/MS	15 (12–18)	Abia et al. (2013)
Congo	Bean	30 (27)	TLC	185.2 (12.5–273.2)	Mulunda et al. (2013)

Abbreviations: nd, not detected; NS, not stated.

**TABLE 4:** Summary of zearalenone occurrence in beverages.

Country	Product	No. of samples	Detection method	Detection range (μg/L)	References
Nigeria	Fermented melon	25 (8)	HPLC	33 (21–45)	Adekoya, Njobeh, et al. (2017) and Adekoya, Obadina, et al. (2017)
	Fermented locust bean	8 (5)	HPLC	18 (11–33)	Adekoya, Njobeh, et al. (2017) and Adekoya, Obadina, et al. (2017)
	Fermented African oil bean	13 (4)	HPLC	72 (39–117)	Adekoya, Njobeh, et al. (2017) and Adekoya, Obadina, et al. (2017)
Nigeria	Kumu	–	LC–MS/MS	0.2	Ezekiel et al. (2015)
	Pito	–	LC–MS/MS	0.2	
Cameroon	Maize beer	14 (12)	LC–MS/MS	17 (1.6–35)	Abia et al. (2013)
	Dagwa	8 (8)	LC–MS/MS	32 (6–57)	

### 9.3 Occurrence of ZEA in animals and animal products

Apart from grains, a few studies also investigated the occurrence of ZEA in animals and animal products in Africa, where it was reported not to be as frequent as in grains. For instance, in South Africa, the mycotoxin survey in red meat from rural subsistence farmers and registered abattoirs by van Deventer et al. (2021) reported no ZEA contamination in all tested samples (LOD; 20 μg/kg). Meanwhile, in Zambia (another Southern African country), Gonkowski et al. (2018) investigated the presence of ZEA and its analog products in 27 sun‐dried Kapenta fish collected from three cities and found ZEA and α‐ZEA in all samples with concentration levels ranging from 27.2 to 53.9 μg/kg and <3.0 to 71.1 μg/kg, respectively, 66.7% tested positive for β‐zearalenone with levels ranging from <12 to 59.8 μg/kg (Gonkowski et al., 2018).

In the West African subregion, 33% of 108 dried beef samples in Nigeria tested positive for α‐zearalenone with concentration levels ranging from 47.6 to 167.34 μg/kg (Dada et al., 2020). ZEA and β‐zearalenone were, however, absent.

### 9.4 Zearalenone in water sample

Recently, various studies have quantified ZEA in agricultural and domestic water sources. This may present a potential contamination route for plants through radical uptake and humans through drinking and cooking with ZEA‐contaminated water (Agnieszka et al., 2012; Rolli et al., 2018). An analysis of water samples from Zambia showed the presence of ZEA in three out of four water samples from lakes, with values ranging from <2.0 to 18.0 ng/L (Gonkowski et al., 2018).

## 10 REPORTED EXPOSURE ASSESSMENT AND RISK CHARACTERIZATION OF ZEARALENONE IN AFRICA

Generally, there are two pathways for measuring human dietary exposure to ZEA. The First and most widely used method is the direct exposure pathway, where dietary exposure evaluation of ZEA combines household consumption data and the concentration of ZEA in food products and expressed as nanogram/kilogram body weight/day. The Food and Agriculture Organization of the United Nations (FAO) and the Joint Expert Committee on Food Additives (JECFA) under the World Health Organization (WHO), as well as the European Food Safety Authority (EFSA), have developed various guidelines for dietary data collection including living standards monitoring survey, household income and expenditure surveys, and National household budget survey.

Using this method, high exposure risks have been reported by some countries in Africa. Consumers in the savannah zone of Nigeria were reported to have a high risk of exposure to ZEA with % tolerable daily intake (TDI) of 395.6, 158.3, and 66 which were 1582, 633, and 264 times higher than the tolerable daily intake of 0.25 μg/kg/bw·day (Adetunji et al., 2017). A study on the occurrence of ZEA in food and dietary exposure of the population to ZEA was assessed in Algeria by Mahdjoubi et al. (2020). Mean ZEA exposure in the adult population through maize was lower (0.08 kg body weight per day; TDI 31.97%) compared to wheat (0.85 kg b.w. per day; TDI 341.4 5).

The second pathway to assessing ZEA exposure is the indirect method. This approach uses biomarkers such as urine, breast milk, and serum. Across the African continent, few studies on the lactational or maternal transfer of ZEA to infant and birth outcomes are available. For Western Africa, Braun et al. (2022) evaluated the association between the transfer of ZEA from food to breastmilk and infants in Nigeria. ZEA was detected in lactating mothers' meals (0.1–33 μg/kg) and urine (11–1142 ng/L) but was not detected in breast milk. However, urine samples from exclusively breastfed infants contained ZEA ranging from 8.6 to 983 ng/L (Braun et al., 2022). Similarly, in Nigeria, Ezekiel et al. (2022) demonstrated that urine samples of exclusively breastfed infants (*N* = 23) contain ZEA ranging from 17 to 784 ng/L, while ZEA could not be detected in breast milk consumed by exclusively breastfed infants (*N* = 22).

Recently in Eastern Africa, Mesfin et al. (2023) evaluated mycotoxins in the breast milk of 138 lactating mothers in Ethiopia and reported that ZEA (LOD = 0.66, LOQ = 4.38) could not be detected. However, another study conducted in Ethiopia that determined the association between ZEA in pregnant women and birth outcomes showed that 50.9% (295) out of 579 serum samples of a pregnant woman contained ZEA with a concentration of up to 9 ng/mL (Tesfamariam et al., 2022). Also, 33.9% (196), 42% (243), 65.3% (378), and 64.6% (374) were positive for zearalenone, alpha zearalenol, beta zearalenol, beta zearalanol at concentrations up to 15.6, 10.5, 13.2, and 12.9 ng/mL, respectively; however, the study found no significant association between ZEA, and its derivative compounds exposures, and birth outcomes (Tesfamariam et al., 2022).

In Southern and Central Africa, Shephard et al. (2013) evaluated ZEA exposure levels in 54 adult females from South Africa using urine samples: ZEA was detected in all the urine samples with a mean level of 0.529 ng/mg creatinine, while 92% and 72% of the samples contained alpha and beta zearalenol with mean concentrations of 0.614 ng/mg creatinine and 0.702 ng/mg creatinine, respectively. In Yaoundé, Cameroon, Abia et al. (2020) collected and analyzed 89 Cameroonian adults' urine for various *Fusarium* mycotoxins; the authors observed that ZEA and its phase I metabolites were the most frequently detected mycotoxins (82% of the sample analyzed contained detectable quantities of ZEA, alpha zearalenol, and beta zearalenol) with two samples exceeding the EU TDI.

Being the major staples of many African communities, cereal and legumes, particularly maize and sorghum, are frequently used as complementary foods for infants and young children. However, the dietary exposure assessment for ZEA reported from different countries in Africa is based on average intake and body weight for adults. This assessment may not represent the true exposure levels for infants and young children.

## 11 INNOVATIVE STRATEGIES TO REDUCE RISK LEVELS OF ZEARALENONE IN AFRICA

### 11.1 On‐field preventive methods

There is an urgent need to transform African indigenous food systems to respond to the present increased occurrence of mycotoxins in food grains. It has been established that the first step in mycotoxin management is to prevent fungi infestation, that is, production systems should incorporate elements and activities that will prevent fungi infestation. Providing agricultural education and technologies aimed at changing farmers' pre‐ and postharvest practices, by using demonstrations (e.g., proper fertilization and fungicide use, with an explanation of their impact on agriculture) may help reduce ZEA occurrence and improve mycotoxin levels in the African food supply chain (Njeru et al., 2019; Visser et al., 2020). That is, scale‐up education on the use of resistant cultivars, appropriate soil amendment methods, proper weed management, and harvesting techniques should be facilitated. Unfortunately, commercially available maize cultivars in Africa have not been tested for specific resistance to *Fusarium species* (Tembo et al., 2022). Previous studies in South Africa, and Nigeria reported potential resistance of some maize‐inbred lines to *F*. *verticillioides* and fumonisin accumulation (Olowe et al., 2015; Small et al., 2012). No studies have so far evaluated maize cultivars from Africa for ZEA resistance.

#### 11.1.1 Impact of soil amendments on *Fusarium* species and zearalenone concentration

Being a typical soil‐borne pathogen, *Fusarium* species survive on or within infected soil and crop residues, which remains the leading cause of grain contamination before harvest. Consequently, using soil amendments to change the microenvironment of soil should be preferred to direct unamended soil. Biochar though more expensive than common fertilizers (Latawiec et al., 2019) can reduce *Fusarium* infestation and expression in crops through its potential to fix carbon and increase soil pH and elementary composition (Akhter et al., 2015; Marra et al., 2018). Recently in Nigeria, Akanmu et al. (2020) found biochar as an effective tool in managing resident *Fusarium verticillioides* and have successfully used biochar from poultry fecal waste and sawdust to reduce disease severity (ear rot) caused by *Fusarium verticillioides* in maize. Activated carbon would serve as an adsorbent to mycotoxins, capable of binding 100% ZEA at 0.1%, 0.25%, 0.5%, and 1% dose levels (Bueno et al., 2005). This adsorption capacity and increased pH explained that biochar and activated carbon not only delayed conidial germination but also carried a high adsorption capacity for mycotoxin (Li et al., 2022).

#### 11.1.2 Contribution of natural extract in controlling *Fusarium* species and zearalenone

The utilization of natural extract is emerging as a possible method to suppress mycotoxigenic fungi growth and their production of mycotoxins in grain under storage. Although a higher a_w_ level favors *Fusarium* growth, antifungal activities of some natural extracts such as essential oils are effective at higher a_w_ levels (Velluti et al., 2004). Olopade et al. (2019) explored the possibility of using montmorillonite clay and/or *Cymbopogon citratus* to decontaminate ZEA in stored grains and observed that 12% *Cymbopogon citratus* reduced ZEA contamination by 98.3% while 12% montmorillonite*–Cymbopogon citratus* mixed showed a 66% reduction of ZEA in for 4 weeks. Ali et al. (2021) suggest that essential oils obtained from *Mentha longifolia* and *Citrus reticulata* can inhibit *F*. *culmorum* growth at 500 μL/mL. Similarly, Sahab et al. (2014) evaluated the antifungal activities of some essential oils extracted from rocket seeds (*Eruca sativa*), rosemary (*Rosmarinus officinalis*) leaves, and tea tree (*Melaleuca alternifolia*) against *Fusarium* isolates obtained from maize. Essential oil from rocket seeds and tea trees exhibited high antifungal activity and completely inhibited all *Fusarium* isolates growth at 0.1% and 0.4% respectively, while rosemary essential oil showed moderated antifungal activity with reduced growth of *Fusarium* isolates (Sahab et al., 2014). At 0.995 a_w_, cinnamon oil, clove oil, lemongrass oil, oregano oil, and palmarosa oil exhibited inhibitory effects on *F*. *graminearum* growth, and their ability to produce zearalenone (Velluti et al., 2004).

#### 11.1.3 Biological‐driven methods to reduce *Fusarium* infection and zearalenone

A good number of microbial species have demonstrated their ability to counter the growth and ZEA excretion in toxigenic *Fusarium* species. A study by Shude et al. (2021) using antagonist yeast species isolated from leaf, flowers, anther, and/or stem of cereals crops, and weed plants exhibited varying inhibitory effects on the mycelial growth of *Fusarium graminearum*. Most of the yeast antagonists (87.21%) that inhibited the growth could maintain inhibition until 20 days postinoculation (dpi); however, about 58.33% (7 out of 12) of the antagonist yeast increased ZEA concentrations (Shude et al., 2021). Subsequently, acibenzolar‐s‐methyl was combined with yeast antagonist (*Papiliotrema flavescens* and *Pseudozyma* sp.) to test against *F*. *graminearum* on spring wheat: it was observed that the combination of yeast and acibenzolar‐s‐methyl treatment effectively reduced *Fusarium* head blight severity, and deoxynivalenol concentration compared to the sole treatments (Nothando et al., 2021; Shude et al., 2022).

Another study conducted by Debbi et al. (2018) explored the potential of using *Trichoderma* spp. from Algeria as a biocontrol agent against *F*. *oxysporum*. f. sp. *lycopersici* (FOL), and *F*. *oxysporum* f. sp. *Radices lycopersici* (FORL) showed that *T*. *ghanense* and *T*. *asperellum* might reduce the severity of crown and root rot and *Fusarium* wilt diseases by 53.1%, and 48.3%, respectively.

### 11.2 Primary processing methods to reduce zearalenone concentration

#### 11.2.1 Cleaning and sorting

Primary intervention methods such as cleaning and sorting of damaged, discolored, and shriveled kernels reduce *Fusarium* toxins in grains (Pascale et al., 2022; Schaarschmidt & Fauhl‐Hassek, 2021). In many African countries, such as South Africa, Ghana, Benin, Nigeria, Malawi, and Tanzania, grains are customarily hand‐sorted and cleaned before domestic and industrial use. However, this process is tedious and difficult to apply in medium‐to‐large‐scale food processing industries. For practical purposes involving high production volumes, mechanized tools and ultraviolet light have been used to segregate ZEA‐contaminated products. Aoun et al. (2020) explored and developed a low‐cost sorting tool (Dropsort device) that can be used in Africa for reducing mycotoxin levels in grains. Though not ZEA, the dropsort, a low‐cost sorter that separates grains based on kernel bulk density and 100‐kernel weight, combined with size sorting was more effective in reducing fumonisin (another *Fusarium* mycotoxin) concentration to under 2 ppm, but could not reduce aflatoxin levels in maize grain to under 20 ppm (Aoun et al., 2020). Although the DropSort accepted fraction had significantly higher 100‐kernel weight and kernel bulk density than the rejected fraction, the technology only or in combination with visual sorting had up to 19% rejection rate, and this may hinder its acceptability in Africa, particularly with commercial traders since volume is the commonly accepted method in trading than weight. Cleaning grains (removal of dust, coarse, small, broken, shriveled, and low‐density kernels) have been tested and proven to be capable of eliminating 67%–87% of ZEA concentration in maize (Pascale et al., 2022).

#### 11.2.2 De‐hulling

De‐hulling is a widespread food processing technique used in Africa and has successfully been used to reduce mycotoxin concentrations in finished food. For zearalenone, the toxin is largely restricted to the outer layers of grain, and therefore, if partitioned into various fractions, including germs, bran, and coarse and fine grits may reduce human and animal exposure to ZEA (Brera et al., 2006; Habschied et al., 2011; Schwake‐Anduschus et al., 2015). In Malawi, Njombwa et al. (2020) evaluated occurrence levels of ZEA in dairy cattle concentrate feed and observed that 75% (83 out of 111) of corn bran tested positive for ZEA with minimum, maximum, and median concentration levels of 100, 2400, and 240 μg/kg, respectively. In South Africa, corn bran, corn flour, corn germ, and corn grits were reported to be contaminated with ZEA at mean levels of 245.6, 31, 29.8, and 8.6 μg/kg, respectively, compared to the mean value of 93.4 μg/kg for whole maize (Burger et al., 2013). The major limiting factor to this control technique in Africa is that the hull is used as a component in animal feeds. This may increase animal exposure risk and invariably human intake of ZEA through the consumption of animal products.

### 11.3 Secondary processing methods to reduce ZEA concentration in grains

#### 11.3.1 Fermentation

Being water‐soluble mycotoxins, ZEA concentration can be partially reduced during fermentation or in an alkaline solution. Under alkaline conditions, the lactone ring of ZEA (Figure 1) is opened to form water‐soluble salt. The transfer of ZEA into water used for soaking or fermentation is effective at elevated pH. African indigenous fermentation process, mainly as spontaneous or in most cases the addition of yeast, is capable of reducing ZEA levels in traditionally fermented food and beverages. In Nigeria, Ezekiel et al. (2015) assessed the levels of ZEA in *Kunu*‐*zaki*—a traditional fermented nonalcoholic maize‐based beverage, and *pito*—a traditional fermented alcoholic drink made from sorghum. It was observed that the traditional fermentation process used in preparing these drinks can reduce ZEA levels up to 76.2% and 94.8% for *kuku*‐*zaki* and *pito*, respectively. Previously in Botswana, Nkwe et al. (2005) analyzed sorghum malts and their corresponding wort and beer samples for ZEA levels; the traditional wort (90 μg/kg) and beer (92 μg/kg) samples contain less ZEA than their raw malted sorghum samples (485 μg/kg). More recently, an effective reduction in the content of ZEA was observed in bread prepared by baking with the addition of yeast, ranging from 14.3% to 35.4% (Podgórska‐Kryszczuk et al., 2022).

#### 11.3.2 Radiation

Radiation is a powerful tool for decontaminating mycotoxins and improving the storability of food is irradiation (gamma radiation, electron beams, and X‐ray). In recent years, ionizing radiation, as a physical, cold process, has been investigated as a method for the degradation of ZEA and its associated fungi in food grains (Calado et al., 2020). In Egypt, Sebaei et al. (2020) examined the reduction of ZEA in grains using gamma radiation and reported that ZEA was more easily degraded in wheat than in maize. In Tunisia, an irradiation dose (gamma radiation) of 3 and 10 kGy was sufficient to reduce 90% of the natural fungal load and 32% of ochratoxin A in sorghum, respectively (Ben Amara et al., 2022). Similarly, Zhao et al. (2019) demonstrated the 90.24% degradation rate of zearalenone using microwave irradiation for 2 min coupled with activated carbon.

#### 11.3.3 Ozone

Ozonation, an advanced environmentally friendly and generally recognized as safe technology, can attack a wide range of microorganisms and natural compounds (Qi et al., 2016; Ribeiro et al., 2021). The process is highly recognized for its ability to generate ozone gas easily from oxygen (pure or from the air) without leaving residues. Ozone has been approved as a safe antimicrobial agent and has enhanced consumer confidence as well as widespread industrial application in the control of industrial pests such as *Fusarium* species (Food and Drug Administration, 2001). It is worth mentioning that ozone application allows water reutilization. This technique has been instrumental in managing *Fusarium* species and ZEA levels in maize products. In a study on maize flour exposure to ozone gas (51.5 mg/L of ozone for up to 60 min), mycotoxin analysis revealed that ZEA concentration was reduced to 62.3% (Alexandre et al., 2019). Similarly, Qi et al. (2016) observed an 86% reduction in naturally contaminated ZEA in maize when exposed to 100 mg/L of ozone for 100 min.

In malt and beer production, Zuluaga‐Calderón et al. (2023) use ozone in the steeping stage to reduce *Fusarium graminearum* incidence from 100% to 47% without affecting the germinative properties. Although the technology has a superior degradation rate for ZEA than electron beam irradiation (Yang et al., 2020), its application increases monounsaturated fatty acids and decreases polyunsaturated fatty acids such as linoleic, oleic, and α‐linolenic fatty acids (Purar et al., 2022; Qi et al., 2016).

#### 11.3.4 Dielectric barrier discharge

Dielectric barrier discharge is another nonthermal treatment technology employed to degrade mycotoxins in food. The technology has been tested to degrade up to 98.28% of ZEA in food products at 50 KV for 120 s (Huang et al., 2022). Zheng et al. (2023) evaluated the impact of dielectric barrier discharge cold plasma on ZEA degradation in maize and observed a 56.57% degradation of ZEA at 50 KV for 120 s with an increase in the fatty acid composition and reduced crude fiber content. Dielectric barrier discharge treatment for ZEA degradation is more efficient in liquid food products (Feizollahi & Roopesh, 2021). Furthermore, dielectric barrier discharge generated from 85% Ar + 15% O_2_ resulted in higher degradation of ZEA compared to N_2_ (Feizollahi & Roopesh, 2021).

## 12 RECENT PROGRESS IN THE USE OF INDUSTRIAL ADSORBENTS

Different industrial adsorbents have been studied at the laboratory level and have been proposed for use in the elimination of zearalenone at the industrial scale level, with temperature playing a critical role in the quantity and maintenance of ZEA. Activated carbon has proven to be effective in eliminating more than 83% of ZEA during the bleaching process of corn oil at 70°C, although excessive temperature (above 100°C) could cause adsorbed ZEA to desorb from activated carbon (Hu et al., 2023). Du et al. (2023) also reported that the metal–organic framework absorbents exhibited sufficient efficacy in removing more than 83.3% of ZEA in vegetable oil within 30 min. Ghafari et al. (2022) found that silica nanoparticles could remove about 92.1% of ZEA in contaminated sunflower oil (Ghafari et al., 2022).

Commercially available inorganic and organic mycotoxin adsorbents such as S‐Mont, Minazel® plus, and Mycosorb® patented and registered as feed quality enhancers have demonstrated their efficacy in alleviating the harmful effect of ZEA on livestock including pigs (Nesic et al., 2008).

## 13 CONCLUDING REMARKS AND FUTURE RESEARCH

**FIGURE 3:**
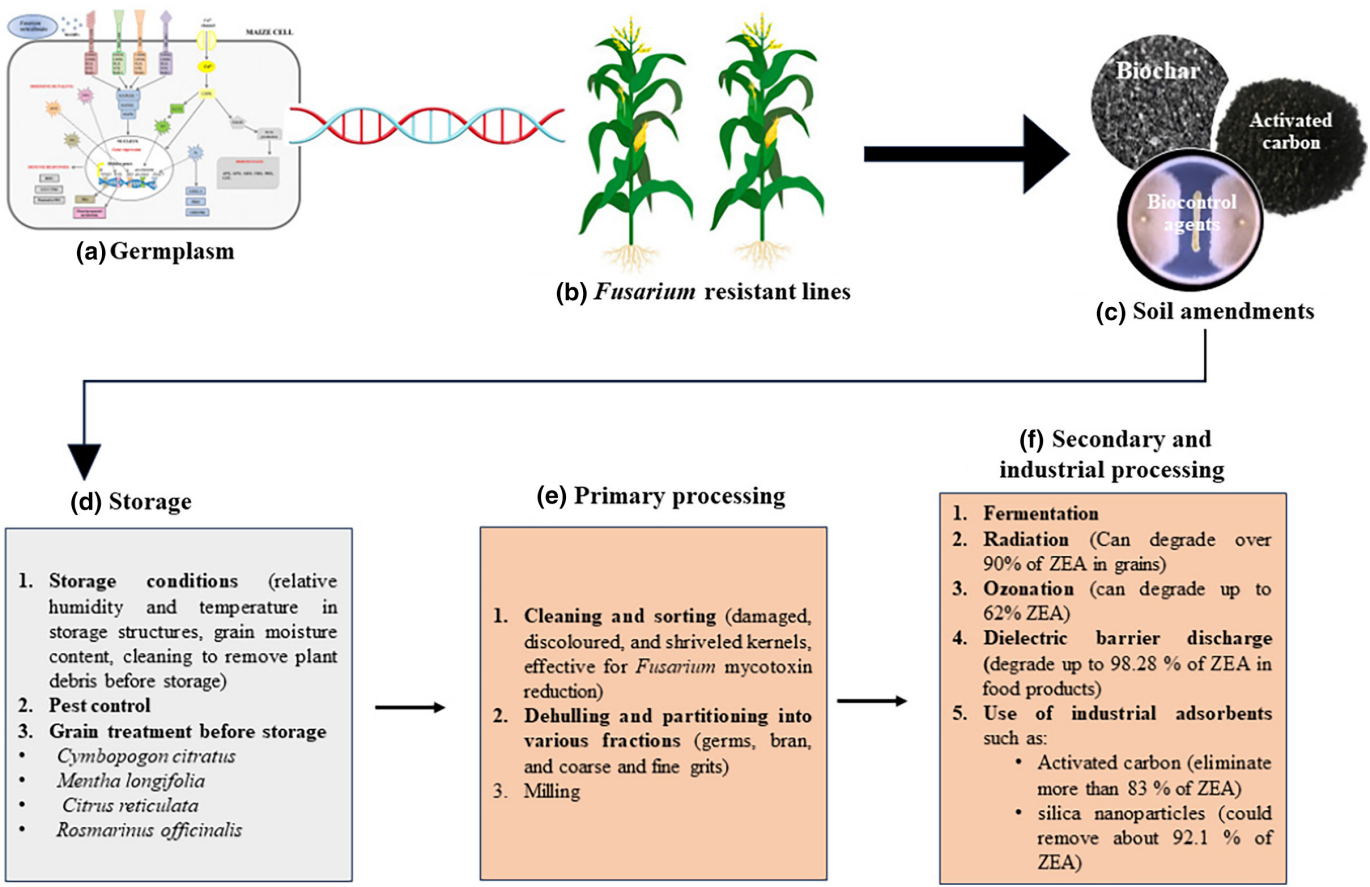
Reported methods used to control *Fusarium* infestation and ZEA concentration in grains and processed food. (a) Hypothetical model explaining phytohormone‐mediated molecular mechanisms in maize plant defense response against *Fusarium verticillioides* (Lanubile et al., 2017), (b) Integration of various molecular tools to develop resistant maize line, (c) using soil amendments to change the microenvironment of soil, (d, e, and f) various postharvest methods employed to reduce ZEA levels in grains and processed foods.

1. Although ZEA has a severe impact on grain quality and the economic well‐being of livestock and humans, it is clear that few studies have evaluated its presence in agricultural produce with not much awareness of the toxin in Africa.
2. Given that climate change patterns are widely evident in Africa with the projected increase in atmospheric CO_2_ concentration and relative humidity, which flavors *Fusarium* species growth and ZEA production, little progress is made in understanding the impact of this changing climate with the recently higher levels of ZEA in Africa. Overall, *F*. *verticillioides* and *F*. *graminearum* were the most commonly isolated species in all the subregional blocks (northern, eastern, western, southern, and central Africa). Understanding plant stress responses, enabling plants to withstand the changing environmental conditions, is essential to address and prevent the *Fusarium* species activities.
3. Preventive and mitigation efforts evaluated and applied to control *Fusarium* species on‐field and in storage as well as ZEA production so far are insufficient and need future investigation (Figure 3).
4. Most countries in Africa do not know the status of ZEA in their food supply chain, and there are limited regulations (only in South Africa and Morocco) to control ZEA occurrence in maize and other cereal crops.
5. Some indigenous food processing techniques used in Africa have proven to have an inherent effect in reducing ZEA levels in processed maize foods; however, not much attention has been given to these techniques to further optimize these processing techniques for ZEA elimination (Figure 3).
6. Although nonthermal food processing technologies have exhibited their power to effectively eliminate a large number of different mycotoxins, including ZEA in processed foods, regulations for its use in mycotoxin management are limited. As such more research is needed to assess the safety of these technologies (ozone, radiation, dielectric barrier discharge) with mycotoxin management in the storage and processing of food, and to enhance consumer acceptability.

## AUTHOR CONTRIBUTIONS

**Abdul Rashid Hudu:** Conceptualization (lead); data curation (equal); methodology (lead); writing – original draft (lead). **Francis Addy:** Data curation (equal); supervision (equal); writing – review and editing (equal). **Gustav Komla Mahunu:** Data curation (equal); writing – review and editing (equal). **Abdul‐Halim Abubakari:** Data curation (equal); writing – review and editing (equal). **Nelson Opoku:** Data curation (equal); supervision (equal); writing – review and editing (equal).

## CONFLICT OF INTEREST STATEMENT

The authors declare no conflicts of interest.

## Data Availability

Data presented in this study are contained in the article.
